# Comparative Assessment of the Antioxidant Activities among the Extracts of Different Parts of *Clausena lansium* (Lour.) Skeels in Human Gingival Fibroblast Cells

**DOI:** 10.1155/2020/3958098

**Published:** 2020-10-07

**Authors:** Tingting Zhu, Wenjian Zuo, Jia Yan, Pan Wen, Zhisheng Pei, Huiyong Lian, Hyeong-Cheol Yang

**Affiliations:** ^1^Department of Food Science and Engineering, School of Food Science and Engineering, Hainan Tropical Ocean University, 1 Yucai Road, Sanya 572022, China; ^2^Department of Dental Biomaterials Science and Dental Research Institute, School of Dentistry, Seoul National University, 28 Yeonkun-dong, Chongro-ku, Seoul 110-749, Republic of Korea

## Abstract

*Clausena lansium* (Lour.) Skeels (wampee) is an outstanding natural plant with medicinal properties. The aim of this study was to compare the cytoprotective effects of four parts of wampee under oxidative stress. The aqueous extracts of leaf, peel, pulp, and seed were tested for the proliferation effects on human gingival fibroblast (HGF) cells and the protective effects in the hydrogen peroxide-induced HGF model. Furthermore, the total glutathione assay and identification of rutin by high-performance liquid chromatography were carried out to attempt to determine whether the cytoprotective effects were related to the total glutathione (GSH) stability and rutin content. The results showed that all of the extracts had no cytotoxicity to HGF at tested concentrations ranging from 50 to 5000 *μ*g/ml during 24 h, and the leaf, pulp, and seed extracts increased proliferation of HGF at relatively high concentrations. All the extracts except for the seed extract significantly decreased the production of reactive oxygen species, and the peel extracts exhibited the most effective antioxidant effect. The leaf extract had the highest anticytotoxicity and GSH stabilization effect in the HGF challenged with hydrogen peroxide. In addition, the relative content of rutin in peel and leaf extracts was higher than that in pulp and seed. The results of GSH assay and rutin identification suggest that different cellular protective effects among the four parts of wampee are partially related to the GSH stabilization and rutin content. These findings provide a scientific basis for the antioxidant effect-related biological activities of wampee extracts.

## 1. Introduction

Wound repair is a series of events involving multiple pathophysiological reactions, including inflammation, proliferation, and remodeling [1], and the antioxidant activity has an important role in the whole healing process. At the beginning of the inflammation stage, reactive oxygen species (ROS) are released into the surrounding environment by the stimulated neutrophils to protect the body against infection; however, when the ROS are present in an excess amount, for example, in the cases of aging, a stressful environment [2], or chronic inflammatory ailments [3–5], overproduced ROS may exhaust the endogenous antioxidants, such as glutathione (GSH), further speeding up the cellular aging process [6], which consequently results in impairing wound healing and tissue regeneration [7]. Antioxidant treatment can decrease the ROS accumulation to allow ROS to play a regulatory role in wound healing process effectively. Rutin, which is known as an antioxidant substance of plant origin, has been reported to be as a kind of supplement to promote wound healing [8].

During the proliferative stage, fibroblast cells are the main players with the function to secrete growth factors and produce collagen, which is necessary for wound repair. Moreover, ascorbic acid, one of the representative antioxidants, increases collagen synthesis even in elderly fibroblasts [9], which may enhance the healing process by leading contraction of wound and reducing wound size. At the last remodeling stage, matrix metalloproteinases (MMPs) play an important role in this phase. Topical antioxidant mixture was reported to downregulate MMP-9 to protect basement membranes [10]. Although the wound healing process of the oral mucosa is similar to that of skin, oral tissue is often threatened by bacterial infections and chewing stimulation due to the particular characteristics of oral environment. In addition, in the case of gingival graft surgery with significant tissue loss in the donor's palate, it is important to find some ingredients that enhance wound healing in the oral cavity. Based on the correlation between antioxidants and wound healing mentioned above, antioxidants seem to be a useful strategy for wound healing by removing the inflammatory cell-induced ROS [11].

*Clausena lansium* (Lour.) Skeels or its common name, wampee, belongs to the family Rutaceae, which is commonly distributed throughout Southern China, especially in the Hainan, Guangdong, Guangxi, and Fujian provinces and occasionally in India, Sri Lanka, and the United States (Hawaii and Florida) [12]. The fruit of wampee ripens from May to July with a similar appearance to grape. The fruit of wampee can be eaten directly or served in preserves and even cooked with meat [13]. The leaves, peel, bark, and pulp of wampee can also be used as a traditional medicine to treat diverse ailments, including asthma, cough, bronchitis, viral hepatitis, and dermatological and gastrointestinal diseases in folkloric systems in different regions [2, 14–17], which might be contributed by the bioactive phytochemical constituents such as amides, flavonoids, coumarins, lactams, carbazole alkaloids, and even the volatile components in the roots, seeds, fruits, stems, and leaves of wampee [18–22].

Recently, studies on wampee have given more attention to its bioactive benefits, and antioxidant activity was one of them. The studies on the antioxidant activity used mostly wampee extracts of organic solvents [2, 13, 15, 23], and little is known about the comparison of cellular antioxidant activities among the aqueous extracts of different wampee parts. Therefore, the aims of this paper were to compare the cytoprotective effects among the pulp, seed, leaf, and peel aqueous extracts of wampee in human gingival fibroblast (HGF) cells under oxidative stress and to monitor the effect of the wampee extracts on promoting oral wound healing for further in vitro and vivo studies.

## 2. Materials and Methods

### 2.1. Chemical Reagents

Cell culture medium and reagents were purchased from Gibco-BRL (Grand Island, NY, USA). Other experimental reagents (2′,7′-dichlorodihydrofluorescein diacetate (DCFH-DA), hydrogen peroxide (H_2_O_2_), and formaldehyde) were obtained from Sigma-Aldrich Co. (Saint Louis, MO, USA), unless otherwise specified. All other chemicals used for analysis were of analytical grade and obtained from Guangzhou Analysis Test Center Keli Technology Development Co. (Guangdong, China).

### 2.2. Plant Material and Preparation of Extracts

The wampee was collected in the fully matured stage in 2018 from Hainan Province, China. The plant was authenticated by Professor Min Li in Hainan Tropical Ocean University (Sanya, China). A voucher specimen (No. 20180630) has been deposited at the herbarium for Research and Development of Natural Plants, Hainan Tropical Ocean University (Sanya, China). After being washed with water and air dried, the seed, peel, and pulp were separated, then continuously lyophilized, and finally powdered with an electric blender. The leaf was air dried and then powdered with an electric blender. The different parts of the wampee (pulp, leaf, seed, and peel powders) were then vacuum packaged and saved at −20°C for further use. The four kinds of wampee powders were suspended in sterilized distilled water at 4°C [24] for 48 h. After centrifuging, the supernatant was filtered through a Whatman filter paper No. 1 to remove any remaining debris, evaporated by a lyophilizer (Lyph Lock6 Model 77595-01; Labconco, Kansas City, MO, USA), and stored at −80°C. The four lyophilized extracts were dissolved in a culture medium and filtered through a 0.22-*μ*m cellulose acetate membrane filter (Pall Corporation, MI, USA) before cell treatment [25].

### 2.3. Cell Culture

Human gingival fibroblasts (HGF) were obtained from Science Cell Research Laboratories (ScienCell Research Laboratories, San Diego, CA, USA). HGF cells were maintained at 37°C in a humidified atmosphere (5% CO_2_/95% air) in Dulbecco modified Eagle medium (DMEM) containing 4.5 g/L glucose, 10% fetal bovine serum (FBS), and antibiotic solution (100 U/mL of penicillin-G and 100 g/mL of streptomycin).

### 2.4. Cell Viability and Proliferation Assay

The cells were seeded in 96-well plates at a seeding density of 0.8 × 10^5^ cells per well until confluent and then reincubated in culture media containing the different parts of the wampee extracts at various concentrations (0, 50, 100, 500, 1000, and 5000 *μ*g/mL). After 24 h, cell viability was assessed with 2-(2-methoxy-4-nitrophenyl)-3-(4-nitrophenyl)-5-(2,4-disulfophenyl)-2H-tetrazolium (WST-8, Dojindo Laboratories, Kumamoto, Japan). The treated cells were incubated in 100 *µ*L of WST-8 solution for 1 h, and then, the absorbance was measured at a wavelength of 450 nm using a plate reader (Sunrise, TECAN, Salzburg, Austria). To examine proliferation, HGF cells were treated with the different extracts of the wampee parts for 48 and 72 h, and the amount of cells was assessed with WST-8.

### 2.5. Evaluation of Hydrogen Peroxide-Induced Cell Cytotoxicity

To examine the effects of the different wampee extracts on the hydrogen peroxide (H_2_O_2_) -induced cytotoxicity, HGF cells were exposed to a combination of different wampee extracts and H_2_O_2_ (0.65 mM or 1 mM). Cells were treated with different wampee extracts at various concentrations (0, 500, and 5000 *μ*g/mL) and 0.65 mM H_2_O_2_ for 18 h. After incubation, the WST-8 assay was performed to determine the number of viable cells.

### 2.6. F-Actin Visualization

HGF cells were seeded on chamber slides (Nunc, Roskilde, Denmark) at a density of 1 × 10^5^ cells/chamber. Cells were treated with different wampee extracts in combination with H_2_O_2_ (0.65 mM) for 18 h before staining. Fluorescence staining for F-actin was carried out using ActinGreen 488 ReadyProbes reagent (Thermo Fisher Scientific, Waltham, MA, USA) according to the manufacturer's instructions. Briefly, the cells were washed with PBS, fixed in fixative solution (4% formaldehyde in PBS) for 10 min at room temperature, permeabilized with permeabilization buffer (0.5% Triton X-100 in PBS) for 5 minutes at room temperature, washed twice with PBS, and stained with ActinGreen 488 ReadyProbes reagent for 30 minutes. Cells were photographed with a microscope (Olympus, Tokyo, Japan).

### 2.7. ROS Measurement

The DCFH-DA is a cell‐permeable dye that becomes fluorescent upon reacting with hydroxyl ROS. ROS was measured as described in our previous paper [26]. Briefly, cells (0.8 × 10^5^ cells/well) grown on 96-well plates were incubated in culture medium containing 20 *µ*M DCFH-DA at 37°C for 20 min, washed once with PBS, and then exposed to 1 mM H_2_O_2_ in the presence or absence of 500 *μ*g/mL wampee extract. The fluorescence of the cells was then measured using a microplate fluorometer (FLUOstar Optima microplate fluorometer, BMG Lab Technologies, Offenburg, Germany) at intervals over the course of 6 h.

### 2.8. Total Glutathione Assay

HGF cells were seeded in 6 cm culture dishes at a density of 2 × 10^5^ cells/dish and treated with the different wampee extracts (1000 *μ*g/mL) in combination with H_2_O_2_ (1 mM) for 3 h. Total GSH was measured using a glutathione assay kit (Beyotime, China) according to the manufacturer's instructions. Briefly, cells were collected with a scraper and then precipitated by centrifugation. Protein remover *M* reagent was added and then mixed well. The cells were lysed using four cycles of freeze-thawing and then centrifuged at 10,000 g for 10 min. The supernatant was mixed with the total GSH test mix buffer. A kinetic profile of the optical density at 405 nm was used to determine the glutathione of the samples.

### 2.9. Rutin Analysis

The identification of rutin in different wampee parts was evaluated by high-performance liquid chromatography (HPLC). The HPLC analysis was carried out on an Agilent Technologies 1260 instrument (Santa Clara, CA, USA) equipped with a Luna C18 column (ø 250 mm × 4.6 mm) and UV detection. Gradient programs were used with the mobile phase consisted of solvent A (water) and solvent B (acetonitrile) as follows: 5% (initial), 5%–30% (30 min), 30%–80% (20 min), and 100% (5 min). At a flow rate of 1.0 ml/min, typically 20 *µ*L portions were injected. The detected wavelength was 254 nm, and the column temperature was maintained at 25°C.

### 2.10. Statistical Analysis

All experiments were performed three times. Data from the three different sets of experiments are expressed as the means ± SD. Significant differences among the test groups (*p* < 0.05) were evaluated with Student's *t*-test or one-way ANOVA followed by Bonferroni's post hoc test.

## 3. Results

### 3.1. Effects of the Different Wampee Extracts on Cell Viability and Proliferation

**Figure 1:**
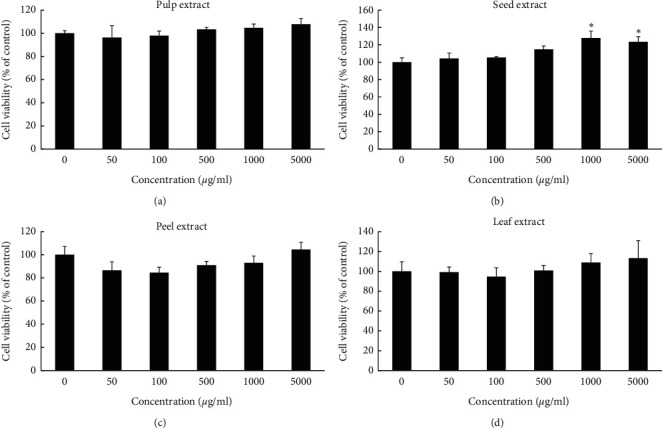
Effects of the four wampee extracts on cytotoxicity in HGF cells: cells were treated for 24 h to investigate the cytotoxicity. Data and error bars indicate the means ± SD of three independent experiments. ^*∗*^ indicates a statistically significant (*p* < 0.05) difference between the control and treated cells by Student's *t*-test.

**Figure 2:**
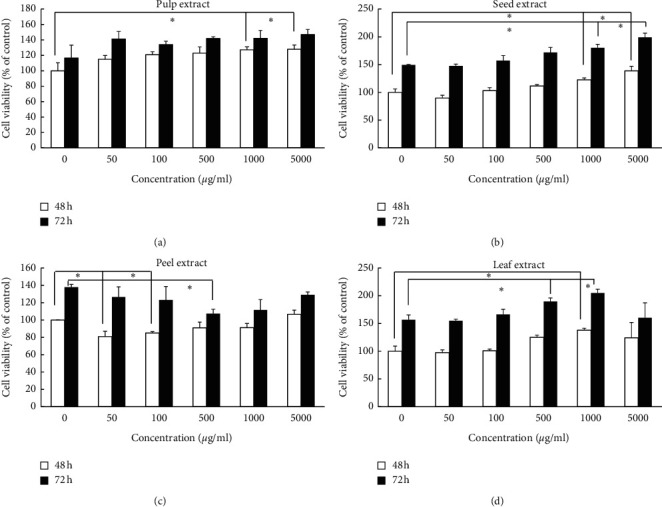
Effects of the four wampee extracts on proliferation in HGF cells: cells were treated for 48 h and 72 h to observe the proliferation ability. Data and error bars indicate the means ± SD of three independent experiments. ^*∗*^ indicates a statistically significant (*p* < 0.05) difference between the control and treated cells by Student's *t*-test.

To investigate whether the aqueous extracts of the different wampee parts influenced the cell viability, HGF cells were exposed to the aqueous extracts at different concentrations (50, 100, 500, 1000, and 5000 *μ*g/mL) for 24 h, and then, the cell viability was measured by WST-8 assay. As shown in Figure 1, all of the aqueous extracts of wampee showed no cytotoxicity at concentrations ranging from 50 to 5000 *μ*g/mL. In addition, wampee seed extract at higher concentrations of 1000 and 5000 *μ*g/mL induced 27% and 23% increase in cell viability, respectively, compared to the control group. Therefore, we further explored the effect of the four extracts on HGF cell proliferation. The seed extract increased the cell proliferation at the 1000 and 5000 *μ*g/mL concentrations during the 48 and 72 h treatment, similar to the trend during the 24 h treatment, demonstrating the continuous effects of the seed extract on the HGF cell proliferation (Figure 2(b)). The pulp extract enhanced cell proliferation at 1000 and 5000 *μ*g/mL statistically significant during 48 h compared to the medium-treated control group (Figure 2(a)). The leaf extract effectively increased the cell proliferation at relatively high concentrations, but it did not last up to the concentration of 5000 *μ*g/mL (Figure 2(d)). The peel extract decreased the cell proliferation at lower concentrations (Figure 2(c)).

### 3.2. The Cytoprotective Role of the Four Wampee Extracts against H_2_O_2_-Induced Cytotoxicity

**Figure 3:**
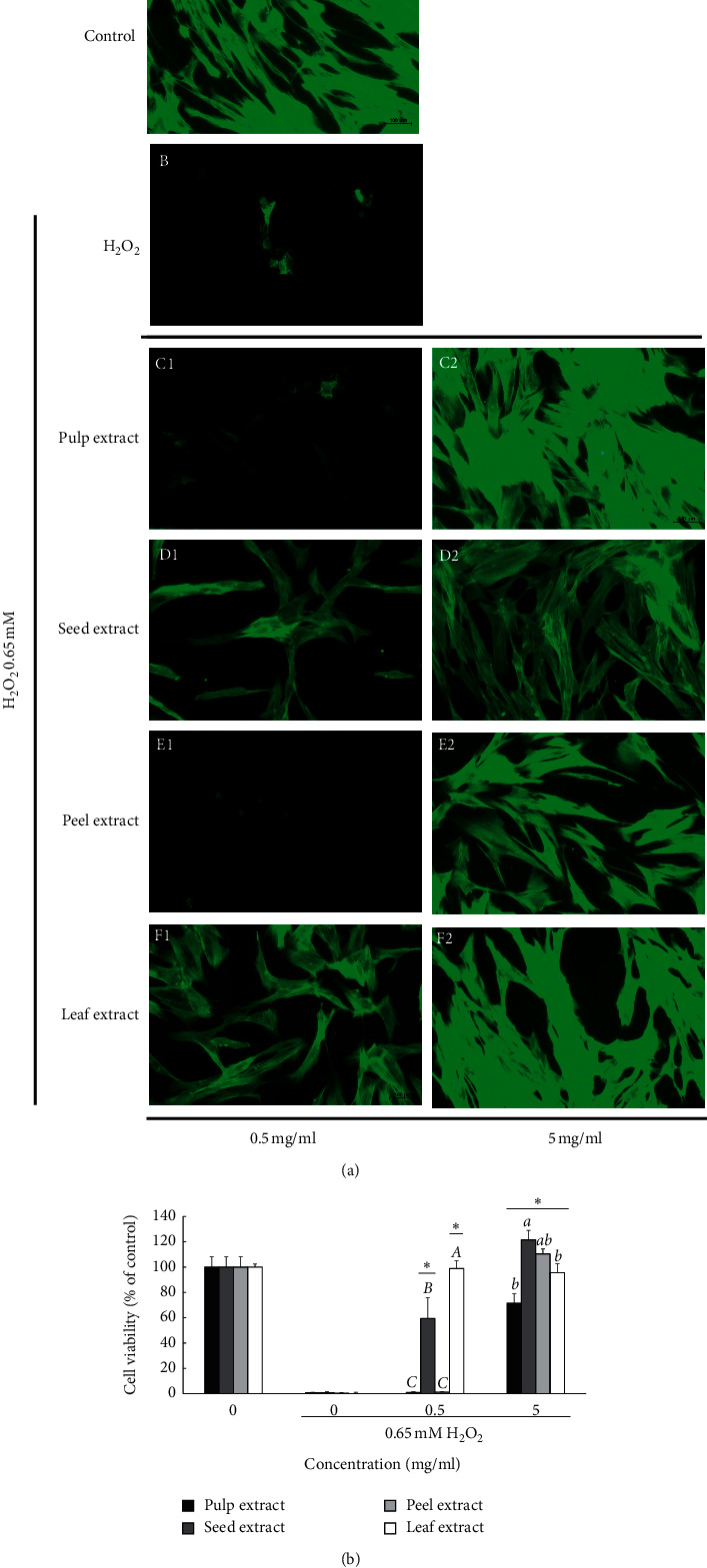
The cytoprotective effect of the different wampee extracts against H_2_O_2_-induced oxidative stress: cells were co-treated with wampee extracts (0.5 and 5 mg/ml) and 0.65 mM H_2_O_2_ for 18 h and then the effects of the wampee extracts on H_2_O_2_-induced cytotoxicity (B) and fluorescence images of F-actin images with 200x magnification (A) were operated. ^*∗*^ indicates a statistically significant (*p* < 0.05) difference between the H_2_O_2_-treated cells and wampee extract-co-treated cells by Student's *t*-test. The cytotoxicity of HGF cells treated with the same concentration of wampee extracts was grouped alphabetically by one-way ANOVA analysis (*p* < 0.05).

The four different wampee extracts effectively protected the HGF cells against H_2_O_2_-induced cytotoxicity. As shown in Figure 3(b), compared with the pulp and peel extract groups, the cytoprotective effect was significantly demonstrated in the seed and leaf extract groups at a concentration of 0.5 mg/mL, for which about 59% and 98% of the cells were recovered, respectively. At 5 mg/mL, the seed and peel extract treated groups were fully recovered, and about 71% and 95% of the cells were recovered in the pulp and leaf extract group, respectively.

The cytoskeleton has an important role in maintaining the cell morphology and in migration. F-actin was stained by phalloidin in HGF cells treated with the different wampee extracts and H_2_O_2_ (Figure 3). H_2_O_2_-treated cells were mostly apoptotic, and F-actin staining was not performed compared to the control group. When combined with the different wampee extracts, F-actin staining showed that the cells recovered from the H_2_O_2_-induced cytotoxicity. Especially in the leaf extract-treated group, the HGF cells had a normal morphology both in the 0.5 and 5 mg/mL in combination with H_2_O_2_ groups.

### 3.3. Antioxidant Effects

**Figure 4:**
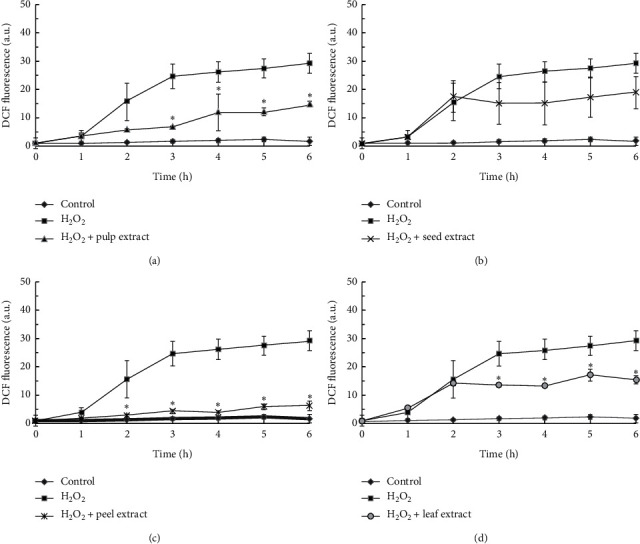
ROS accumulation was measured by DCFH-DA fluorescent dye. Cells were loaded with 20 *µ*M DCFH-DA for 20 min and then washed with PBS and re-incubated with 1 mM H_2_O_2_ and 500 *µ*g/ml of the different wampee extracts. Fluorescence was then measured at regular intervals for 6 h Three independent experiments were performed to obtain the mean fluorescence values and standard deviations. ^*∗*^ indicates a significant difference between the H_2_O_2_-treated and wampee extract-co-treated cells (*p* < 0.05).

To investigate the antioxidant effects of the different wampee extracts, H_2_O_2_-induced hydroxyl radicals were detected in wampee extract-treated cells by using DCFH, which can be oxidized to a highly fluorescent DCF by hydroxyl radicals [27]. Due to HGF's relative strong tolerance to H_2_O_2_ shown in a previous study [26], a relative high concentration of H_2_O_2_ (1 mM) was used in the present study. The HGF cells were treated with the different wampee extracts and H_2_O_2_ in combination, and the peel extract prevented the increase in DCF fluorescence intensity significantly at 2 h, while the pulp and leaf extracts suppressed the DCF fluorescence intensity at 3 h. And there was no statistical significance in the attenuation of DCF fluorescence intensity in the HGF cells by seed extract (Figure 4).

### 3.4. Effects of the Different Wampee Extracts on the Intracellular GSH Level

**Figure 5:**
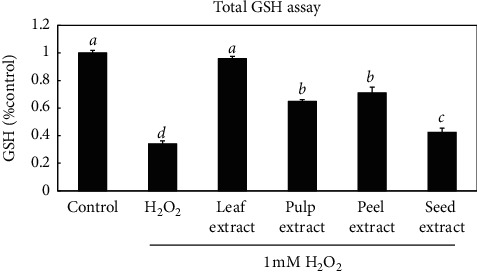
Effects of the wampee extracts on total glutathione. Cells were treated with the different wampee extracts (1000 *μ*g/ml) in combination with H_2_O_2_ (1 mM) for 3 h Total glutathione was measured. Values and error bars indicate the mean glutathione level and standard deviations, respectively. Data were expressed as the means ± SD of three independent experiments. The values were grouped alphabetically by one-way ANOVA analysis (*p* < 0.05).

Glutathione, which can directly scavenge ROS [28], plays an important role in the cytosolic redox system. Thus, we next determined the effects of four kinds of wampee extracts on the intracellular GSH. The amount of total glutathione in HGF cells was ordered as follows: leaf extract > peel extract > pulp extract > seed extract-treated group (Figure 5). Especially, there was no significant difference between the leaf extract-treated group and normal control group. In addition, compared with the only H_2_O_2_-treated group, the decrease of glutathione was more mitigated in the other wampee extracts-treated groups. These results imply that the differences in the degree of GSH depletion was related to the protective effects of four extracts on the HGF cells against H_2_O_2_-induced oxidative stress, especially for leaf extracts.

### 3.5. Rutin Identification among the Different Wampee Extracts

**Figure 6:**
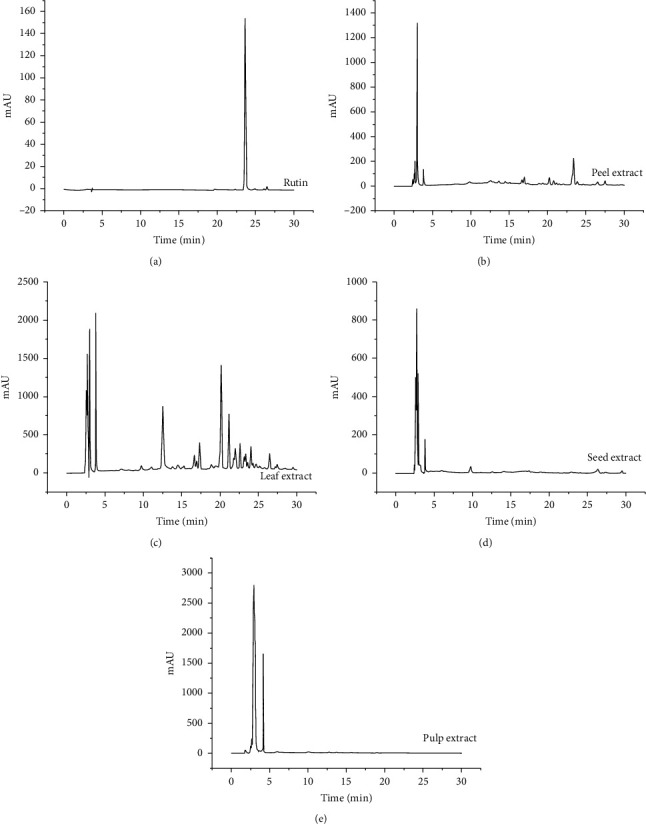
HPLC assay was measured by loading UV indicator with detected wavelength of 254 nm. Gradiert programs were used with the mobile phase consisted of water and acetonitrile. The chromatographic peak of rutin was appeared at about 23.64 min. The value of peak area showed the content of rutin.

Retention time was used in the HPLC analysis to identify the rutin among the four kinds of the wampee extracts. The result showed that a similar chromatographic peak appeared at about 23.64 min, and S/N ratios of all peaks were more than 3, which indicate that the four kinds of wampee extracts contain rutin. Based on peak area, the higher contents of rutin were demonstrated in peel (1.57%) and leaf (0.76%) (Figure 6).

## 4. Discussion

The redox system has an important role in the whole healing process, and an exogenous natural antioxidant would be a benefit for enhancing wound healing [11]. Therefore, in the present study, we observed the antioxidant effects of the aqueous extracts from the peel, pulp, seed, and leaf of wampee in a H_2_O_2_-induced HGF cell model. In addition, the use of aqueous extract can avoid the drawbacks of hydrophobic reagents such as curcumin, which has been reported to have poor aqueous solubility and permeability [29, 30]. As expected, we found that all of the aqueous extracts did not exhibit any cytotoxicity in HGF at the tested concentrations ranging from 50 to 5000 *μ*g/mL during 24 h (Figure 1); furthermore, the leaf, pulp, and seed extracts increased the proliferation of HGF at relatively high concentrations (Figure 2), which is a potential useful aspect for wound healing [31, 32].

And then, H_2_O_2_-induced cytotoxicity in HGF was examined. H_2_O_2_ is also the active ingredient in tooth bleaching agents [33], which has been reported to stimulate irritation, ulceration on the gums [34], and toxic effects on human gingival fibroblasts [35]. Our previous study assessed the catalase activities among MC3T3-E1, human dental pulp cells, HGF, and L929 cells. The highest activity was detected in HGF cells, which explained the reason that HGF cells are more resistant to H_2_O_2_-induced cytotoxicity than other cells, such as human pulp cells [26]. Thus, we used a relatively high concentration of H_2_O_2_ in the present study to assess the effects of wampee extracts on the H_2_O_2_-induced HGF cell model. All extracts significantly attenuated the cytotoxicity generated by H_2_O_2_ (Figure 3), and the greatest protective effect was obtained by the treatment with the leaf extract; moreover, the protective effects were in the following order: leaf extract > seed extract > peel extract > pulp extract. In addition, the F-actin staining, which is required for cell movement and changes in cell morphology, was consistent with the cell-protected effects (Figure 3). The leaf extract maintained the cell morphology and actin staining similar to control cells at both 0.5 and 5 mg/mL concentrations.

Because the four kinds of wampee extracts had a different cell protective ability against H_2_O_2_-induced cytotoxicity, we thought that the antioxidant effect could be the reason for the difference; thus, ROS in treated cells was further examined for the four extracts. Among the ROS, the most lethal radical is a hydroxyl radical, which can be produced with transition metal ions such as iron and copper via the Fenton reaction [36]. Furthermore, DCFH can react directly with the hydroxyl radical but not superoxide or H_2_O_2_. A fluorometric assay with (DCFH-DA) was used to detect hydroxyl radicals in the present study. As shown in Figures 4(a)–4(d), the peel, pulp, and leaf extracts except for the seed extract decreased the hydroxyl radical production significantly when exposed to 1 mM H_2_O_2_. Especially the peel extract almost quenched the DFC fluorescence early on in the treatment time, while the leaf extract did not exert the highest antioxidant ability to deplete hydroxyl radicals. These results indicate that the hydroxyl radical has a limited role in the H_2_O_2_-induced cytotoxicity, partially due to the limitation of the cytosolic labile ions [26].

Because previous studies have indicated that intracellular GSH has a central role in removing cellular H_2_O_2_ in biological systems [11], we investigated whether the different cell protective abilities of wampee extracts against H_2_O_2_-induced cytotoxicity positively correlated to the extent of maintaining intracellular glutathione. The differences in the degree of GSH depletion are shown in Figure 5. All the extracts antagonized the downregulation of GSH compared to that in the only H_2_O_2_-treated group, which may resist an increase in the amount of H_2_O_2_ in the mitochondria and by extension, protect the cells from oxidative stress. Specifically, the leaf extract stabilized the intracellular GSH to the normal level even in the presence of exogenous H_2_O_2_, which consistent with that the leaf extract has the greatest ability among the four extracts to help HGF survive against the H_2_O_2_-induced cytotoxicity.

Zeng et al. demonstrated that flavonoids in the wampee fruit organic extract resulted in the free radical-scavenging and antioxidant activities [37], and other previous study also showed that the scavenging of hydroxyl radicals may be due to the presence of hydrogen-donating flavonoids in extracts [38]. As a flavonoid belonging to the flavonols, rutin is present plentifully in wampee fruits [13] and is a hydrogen-donating antioxidant, which can scavenge free radicals [39–41]. Thus, we further explored the rutin component of the four extracts. As shown in Figure 6, based on the peak area, the rutin in the peel extract represented the highest amount, which confirmed the correlation between the rutin and hydroxyl radical-scavenging activity (Figure 4). In addition, the diversity of phytochemicals in leaf extracts is shown in Figure 6. Based on our current results, the rutin might be a potential candidate, which plays some roles in the effect of wampee on the cell viability and resistance to H_2_O_2_ oxidative stress.

## 5. Conclusions

In this study, we found that the aqueous extracts of wampee had a positive effect on cell proliferation and a protective effect on HGF cells against H_2_O_2_-induced oxidative environment to different degrees. Specifically, the leaf extract showed the highest anticytotoxicity and GSH stabilization effects on HGF challenged with H_2_O_2_. In addition, the peel demonstrated the most effective activity of quenching the DFC fluorescence. Lastly, the leaf, pulp, and seed extracts demonstrated the potential to enhance the proliferation of HGF cells. The results throw a light for using wampee in wound healing, especially in oral environment. Since the leaf, peel, and seed of wampee are usually uneaten and treated as waste, utilization of them has particular advantages in economy benefit and environmental protection. Further research will be required to identify which kind of extracts could be useful in enhancing oral wound healing in in vitro and in vivo studies, and particular attention will be paid on wampee leaf and peel part to increase economic benefits.

## Data Availability

The data used to support the findings of this study are available from the corresponding author upon request.
